# RP gene haploinsufficiency promotes extra sensory organ formation via a threshold effect

**DOI:** 10.1080/19336934.2025.2606496

**Published:** 2026-01-06

**Authors:** Haiwei Pi, Kuan-Han Chen, Hsin Tu, Chung-Wei Hsu

**Affiliations:** a Department of Biomedical Science, College of Medicine, Chang Gung University, Taoyuan, Taiwan; b Graduate Institute of Biomedical Sciences, College of Medicine, Chang Gung University, Taoyuan, Taiwan

**Keywords:** RP gene gene haploinsufficiency, minute mutant, bristles, campaniform sensilla, Xrp1, ribosomal stress, proneural proteins

## Abstract

Ribosomal protein (RP) gene haploinsufficiency is a conserved form of ribosome dysfunction across species and underlies a class of disorders known as ribosomopathies. In *Drosophila*, RP gene haploinsufficiency manifests as the Minute phenotype, characterized by thinner and shorter mechanosensory bristles. The development of both bristles and proprioceptive campaniform sensilla (CS) is initiated by the bHLH proneural proteins Achaete (Ac) and Scute (Sc). By analysing genetic interactions between *ac sc* mutants and *Minute* mutants of varying severity, we identified a novel bristle-promoting effect that occurs only in the strongly affected *Minutes* in which the average bristle length is shorter than a threshold. This threshold-dependent effect also promotes ectopic CS formation in the strong *Minutes*. Transcriptomic analyses comparing the sensory organ – promoting and non-promoting *Minutes* revealed significant differences in stress-response pathways, including differentially elevated expression of the Xrp1–Irbp18 transcriptional dimer. Notably, mutation of *Xrp1* suppresses the ectopic CS phenotype, indicating a positive regulatory role. These findings reveal a previously unrecognized threshold effect in RP gene haploinsufficiency, in which excessive Xrp1 activity promotes supernumerary sensory organ formation, suggesting a compensatory mechanism that modulates neurogenesis under severe ribosomal stress.

## Introduction

Ribosomopathies are a group of disorders caused by mutations in ribosome components or ribosome biogenesis factors [1–3]. Among the most common genetic leisons underlying ribosomopathies is the ribosome protein (RP) gene haploinsufficieny, in which a heterozygous mutation of a single RP gene causes defects. For example, Diamond-Blackfan anaemia (DBA) is primarily caused by RP gene haploinsufficiency, and leads to a selective failure of erythroid differentiation [4,5]. In *zebrafish*, RP gene haploinsufficiency results in developmental delay, small body size, microcephaly, reduced eye size, and haematopoietic defects [6–8].

In *Drosophila*, RP gene haploinsufficiency give rises to the well-characterized ‘*Minute*’ phenotype, marked by thin and short macrochaete bristles. It is believed that the bristle shortening is caused by defect in protein synthesis [3,9]. Of the 88 RP genes in the *Drosophila* genome, mutations in 66 of them are associated with the *Minute* phenotypes [9]. In addition, *Minute* mutants showed prolonged development [9,10]. Previous analyses of *RpS3* allelic series demonstrated that severity of bristle shortening and developmental delay is proportional to the reduction of *RpS3* mRNA [11].

*Minute* cells are selectively eliminated when surrounded by the wild-type cells in mosaic tissue, a process known as cell competition. This process is driven by the upregulation of the stress response transcription factor Xrp1 in *Minute* cells [12–16]. Xrp1 is a bZIP transcription factor that forms heterodimers with Irbp18 to activate stress-induced transcriptional program. Besides RP gene haploinsufficiency, Xrp1 is also induced by various forms of cellular stress including DNA damage by X-ray irradiation, endoplasmic reticulum stress, and spliceosome dysfunction [17–20].

Bristles, including macrochaetes and microchaetes, are tactile mechanosensory organs [21]. Their development has long served as a genetic model for studying neurogenesis [22–25]. Each sensory organ arises from a single sensory organ precursor cell that is determined by the proneural bHLH transcriptional factors [26–28]. The bHLH proneural proteins are conserved across metazoans, acting as master regulators of neural progenitor cell fate determination and differentiation [29,30]. In *Drosophila*, proneural proteins Achaete (Ac) and Scute (Sc) act redundantly to activate the formation of macrochaetes, microchaetes, and the strain-sensing campaniform sensilla (CS) on the wing [28,31].

While *Minute* mutants exhibit a range of phenotypes, including shortened bristles, reduced body size, delayed development, low fertility, poor viability [32,33], and cardiomyopathy [34], their role in regulating neurogenesis remains poorly understood. In this study, by quantifying sensory organ numbers, we discovered that a subset of strongly affected *Minute* mutants, specifically those with shaft length below a defined threshold, paradoxically promotes the formation of additional sensory organs. Transcriptomic comparison between ‘sensory organ-promoting’ and ‘non-promoting’ *Minutes* revealed quantitative difference in the activation of the stress response pathways, particularly in the expression levels of Xrp1-Irbp18 transcriptional dimer. Moreover, *Xrp1* mutation suppressed ectopic CS formation, indicating that Xrp1-dependent stress response plays a positive role in promoting neurogenesis under conditions of severe RP gene haploinsufficiency that cause the stronger bristle-shortening phenotype.

## Materials and methods

### Fly strains

In all experiments, *w*^*1118*^ flies was used as the wild-type control (*+/+*). The following strains were used in this study:

*ac*^*sbm*^ (BDSC #36539), *RpL10Ab*^*CB02653*^ [35], *RpS24*^*SH2053*^ (BDSC #29511), *RpL14*^*1*^ (BDSC #2247), *RpL18*^*m(3)65F*^ (BDSC #63082), *RpS17*^*6*^ (BDSC #6177), *RpS3*^*2*^ (BDSC #1696), *RpL38*^*45–72*^ (BDSC #6974), *Xrp1*^*M2-73*^ (BDSC #81270), *dpp-Gal4* [36], and *UAS-Xrp1* (FlyORF #F000655). The RP gene heterozygous adults were derived by crossing the balanced stocks with *w*^*1118*^ females. For genetic interaction between *Minutes* and *Xrp1 (*or *ac sc)*, *Xrp1*^*M2-73*^ (or *ac*^*sbm*^) homozygous females were crossed to the balanced, *Minute* stocks to obtain the double heterozygous progenies. The full genotypes of the flies are listed in the Supplemental Table 3.

### Immunostaining

Pupal wings at 24 h APF (29 °C) were prepared by first removing the pupal cases in 1× PBS, followed by fixing the intact pupae in 4% paraformaldehyde for 1 h at room temperature. After fixation, the internal pupal membranes covering the wing tissue were carefully removed. The exposed wings were then refixed in 4% paraformaldehyde for 15 min. Samples were permeabilized in 0.3% Triton X-100 in PBS (PBST) and incubated with primary antibodies overnight at 4°C. Secondary antibodies were applied for 2 h at room temperature. The primary antibodies used were mouse anti-Elav 9F8A9 (DSHB, 1:50), mouse anti-Futsch 22C10 (DSHB, 1:50), and goat anti-Su(H) dC-20 (Santa Cruz, 1:500). Immunofluorescence images were acquired using a Zeiss LSM 780 confocal microscope.

### Rna preparation and transcriptomic analysis

Total RNA was extracted from 70 pairs of wing discs dissected from the wandering third-instar larvae by TRIzol reagent (Invitrogen). For each sample, 200 ng of purified mRNA was used for cDNA library construction by NEBNext UltraTM RNA Library Prep Kit. Three biological replicates were prepared for *w*^*1118*^, and two replicates each for *RpS17*^*+/-*^ (*RpS17*^+/-^) and *RpL14*^*+/-*^.

Raw sequencing data were analysed by STAR (v2.7.3a) two-pass mapping strategy to align the raw FASTQ reads against the *Drosophila* melanogaster reference genome (BDGP6.46). Differentially expressed genes (DEGs) between each *Minute* mutant and the control (*w*^*1118*^) were defined as those with ratio > 1.5-fold (50% increase) or < 0.66-fold (34% decrease) in average read counts and a *p* value < 0.01 (Figure 3A).

To identify DEGs between *RpS17*^*+/-*^ and *RpL14*^*+/-*^ (Figure 3B), the following selection criteria were used:

Upregulated genes: average expression ratio > 1.5-fold (*p* < 0.01) in *RpS17*^*+/-*^ vs. *w*^*1118*^, > 1.1-fold (*p* < 0.05) in *RpS17*^*+/-*^ vs. *RpL14*^*+/-*^, and > 0.9-fold in *RpL14*^*+/-*^ vs. *w*^*1118*^.

Downregulated genes: average expression < 0.66-fold (*p* < 0.01) in *RpS17*^*+/-*^ vs. *w*^*1118*^, < 0.91-fold (*p* < 0.05) in *RpS17*^*+/-*^ vs. *RpL14*^*+/-*^, and < 1.1-fold in *RpL14*^*+/-*^ vs. *w*^*1118*^.

Xrp1-dependent genes in *RpS3*^*+/-*^ mutant were selected based on the criteria described [16].

### Quantification and statistical analysis

The adult flies for quantification of posterior scutellar macrochaete length, microchaete numbers, and CS numbers were collected during the first seven days of the emergence. After the 7-day collection, adult fly bodies are stored in isopropanol on the next day, followed by dissection and picture-taking. All *Minute* adults were collected from two-to-three replicate crosses.

For quantification of shaft length and bristle numbers, images of the adult notum were captured and shaft lengths were measured using ImageJ. Few ‘broken’ bristles with blunt-end at the tip were observed in the *Minutes,* and they are not included for the quantification.

Microchaete numbers were counted within the dorsal-central (DC) region, defined by the position of the DC macrochaetes as the boundaries.

Campaniform sensilla (CS) numbers were determined by counting the dome-like structure on the wing vein III under a light microscope.

Statistical analyses were performed using unpaired two-tailed *t*-tests. Statistical significance is denoted as follows: *p < 0.05, ** *p* < 0.01, ***p < 0.001.Values with *p* > 0.05 were considered not significant (n.s.).

## Results

### RP gene haploinsufficiency causes ectopic formation of mechanosensory organs via a threshold effect

**Figure 1.**
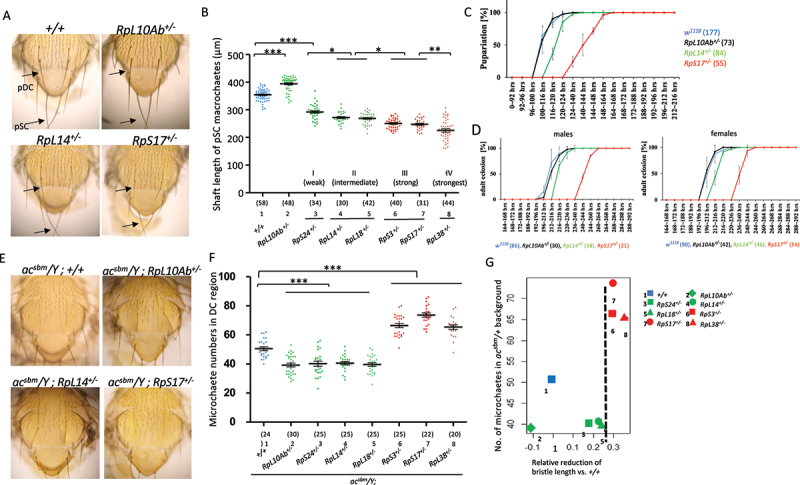
Strong *minute* mutants promote mechanosensory organ formation. (A) Pictures of adult nota. Arrows mark the posterior dorsal-central (pDC) and posterior scutellar (pSC) macrochaetes. (B) Quantification of pSC macrochaete shaft length. *minute* mutants were categorized into four groups by severity of bristle shortening (I-IV). The shaft length was measured using ImageJ. (C and D) developmental timing of pupariation (C) and adult eclosion (D) of *w*^*1118*^, *RpL10Ab*^*+/-*^, *RpL14*^*+/-*^, and *RpS17*^*+/-*^. Sample size was shown in the parentheses. (E) Adult nota of *minute* mutants in *ac*^*sbm*^ hemizygotes. (F) Quantification of microchaete numbers in dorsal-central region. The strong and strongest *minute* mutants increased bristle numbers in *ac*^*sbm*^ mutant, while weaker *Minutes* reduced them. (G) Threshold effect in bristle-promoting activity: only *minutes* with the average shaft length reduced beyond a critical limit (vertical dashed lines) triggered extra bristle formation.

RP gene haploinsufficiency is a common cause of ribosomopathies. In *Drosophila*, it manifests as the *Minute* phenotype, characterized by the thinner and shorter shafts of macrochaetes (large mechanosensory bristles) (Figure 1A, bottom panels). However, the potential roles of RP gene haploinsufficiency in regulating sensory organ formation remain unclear.

To investigate this, we analysed six *Minute* alleles; three from 40S subunit genes (*RpS3*^*+/-*^, *RpS17*^*+/-*^, *RpS24*^*+/-*^) and three from 60S subunit genes (*RpL14*^*+/-*^, *RpL18*^*+/-*^, *RpL38*^*+/-*^) that are randomly selected from available stock centre lines. All six mutants exhibited dominantly shorter and thinner macrochaete shafts compared to the control *w*^*1118*^ (*+/+*) (Figure 1A, arrows; quantified in Figure 1B). Previous analyses demonstrated that bristle shortening and prolonged larval development is proportional to the reduction of ribosome protein mRNA, at least in the case of *RpS3* [11]. Based on the shaft length of the posterior scutellar bristles, these *Minutes* were classified into four severity groups (Figure 1B). Group I (weak): *RpS24*^*+/-*^, the longest shaft among the six *Minute* mutants. Group II (intermediate): *RpL14*^*+/-*^ and *RpL18*^*+/-*^. Group III (strong): *RpS17*^*+/-*^ and *RpS3*^*+/-*^. Group IV (strongest): *RpL38*^*+/-*^, with the shortest shafts. As a non-*Minute* RP control, we included *RpL10Ab*^*+/-*^ which does not produce small-bristle phenotypes in heterozygotes [9]. Although *RpL10Ab* is essential for oogenesis [35], its heterozygotes showed slightly longer bristles than *w*^*1118*^ (Figure 1A and 1B).

Consistent with the severity classification based on shaft length, the larval development time of the strong *Minute RpS17*^*+/-*^ was prolonged much longer (24 hours) than the intermediate *Minute RpL14*^*+/-*^ (4 hours) (Figure 1C). The non-*Minute RpL10Ab*^*+/-*^ showed comparable development timing as the control *w*^*1118*^ (Figure 1C). Delay for adult emergency was observed for *RpS17*^*+/-*^ (24–28 hours) and *RpL14*^*+/-*^ (4–8 hours) but not for non-*Minute RpL10Ab*^*+/-*^ (Figure 1D)

To assess the influence of RP gene haploinsufficiency on sensory organ formation, we examined genetic interaction between the proneural genes *ac sc* and the *Minute* mutations. *ac*^*sbm*^ is a hypomorphic allele affecting both *ac* and *sc* expression [37,38]. In *ac*^*sbm*^ hemizygotes, the numbers of notal microchaete (small bristles) were markedly reduced (comparing *+/+* in Figure 1A to *ac*^*sbm*^*/Y*; +/+ in Figure 1E). As expected, the non-*Minute* (*RpL10Ab*^*±+/-*^), weak *Minute* (*RpS24*^*+/-*^), and intermediate *Minutes* (*RpL14*^*+/-*^ and *RpL18*^*+/-*^) all further decreased microchaete numbers in *ac*^*sbm*^ background (representative figures in Figure 1E, and quantified in lanes 2–5 in Figure 1F, *p* < 0.001 as compared to *+/+*).

Strikingly, the strong *Minutes* (*RpS17*^*+/-*^ and *RpS3*^*+/-*^) and the strongest *Minute* (*RpL38*^*+/-*^) mutants instead increased bristle numbers in *ac*^*sbm*^ mutant (a representative image in Figure 1E; quantified in lanes 6–8 in Figure 1F, *p* < 0.001 as compared to *+/+*).

By comparing bristle number changes in relation to the severity of *Minutes* measured by shaft length reduction, we identified a threshold effect: only the *Minutes* in group III and VI that displayed the reduction of average shaft-length below a certain limit exhibited the microchaete-promoting phenotype (Figure 1G, vertical dashed line). Together with the data showing that the developmental delay is more severe in the bristle-shortening group III than group II, our results reveals that the severe RP gene haploinsufficiency elicits a bristle-promoting response.

### The strong minutes promote ectopic campaniform sensilla formation on the wing vein

In addition to the mechanosensory bristles on the notum, the proneural proteins Ac and Sc also promote the formation of the proprioceptive campaniform sensilla (CS), specialized sensory organs on the wing vein [39]. To examine if the sensory organ-promoting effect of the stronger *Minutes* extends to CS formation, we examined the numbers of CS on wing vein III across different RP gene haploinsufficiency backgrounds.

**Figure 2.**
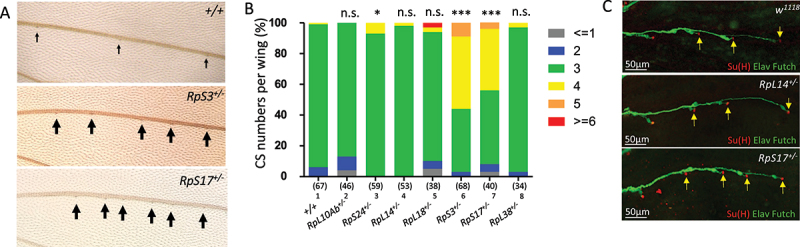
Strong *minute* mutants *RpS3*^*+/-*^ and *RpS17*^*+/-*^ trigger ectopic campaniform sensilla formation. (A) Representative images of adult wing vein iii. Arrows mark the dome-shaped cs. (B) Distribution of cs numbers (2, 3, 4, 5, or 6) per wing among the indicated genotypes: lane 1: wild-type control; lane 2: non-*minute* (*RpL10Ab*^*+/-*^), lanes 3–5: weak/intermediate *minute* mutants; lanes 6–8: strong and strongest *Minutes*. Statistical comparisons are relative to the control. (C) Immunostaining of pupal wings. Arrows indicate the outer support cells of the cs organs marked by anti-Su(H) antibody. There is one extra cs organs (four in total) on the vein iii of *RpS17*^*+/-*^ wing. Dendrites were extended towards and touched the outer cells in each cs organ on the vein iii in all three genotypes.

In wild-type *w*^*1118*^ wings, three CS were consistently present on wing vein III (arrows in Figure 2A and lane 1 in Figure 2B). Notably, the two strong *Minute* mutants, *RpS3*^*+/-*^ and *RpS17*^*+/-*^, significantly increased the numbers of CS organs, often producing four or more per wings (Figure 2A and lanes 6 and 7 in Figure 2B). In contrast, none of the non-*Minute* (*RpL10Ab*^*+/-*^) and intermediate (*RpL14*^*+/-*^ and *RpL18*^*+/-*^) mutants showed a significant increase in CS numbers compared to control (lanes 2, 4, and 5 in Figure 2B). The weak *Minute RpS24*^*+/-*^ even exhibited a significant decrease in CS numbers (lane 3 in Figure 2B).

One exception was observed in *RpL38*^*+/-*^ (lane 8 in Figure 2B), which strongly promoted bristle formation in the notum (Figure 1F) but did not significantly induce ectopic CS. This suggests a possible context-dependent effect of the sensory organ-promoting mechanism in *RpL38*^*+/-*^. The difference to induce ectopic sensory organ formation among the six *Minutes* is unlikely resulted from unique spatial or temporal expression patterns; they all show high expression ( > 50 expression units) in all developmental stages and developmental tissue examined (modENCODE database). Taken together, these findings support a model in which a novel, neurogenic response is triggered by RP gene haploinsufficiency, but only when ribosomal function is reduced beyond a specific severity threshold.

Further examination of the developing CS organs confirmed that extra CS was specified along the wing vein III of *RpS17*^*+/-*^ (Figure 2C, arrows indicate outer cells). Neurons extended dendrite that touched the neighbouring outer cells in all CS organs (Figure 2C), suggesting that the CS organs including the endogenous and ectopic ones, are innervated. In contrast, neither extra outer cells nor neurons were found on the vein III of *RpL14*^*+/-*^ pupal wings (Figure 2C).

### Transcriptomic comparison between RpS17^+/-^ and RpL14^+/-^ reveals differential activation of stress responses

**Figure 3.**
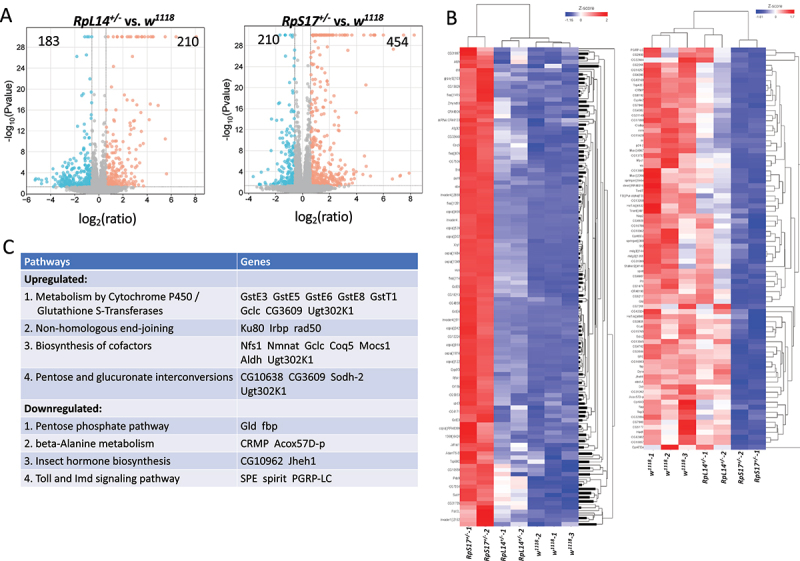
Transcriptomic comparison of the *minutes RpS17*^*+/-*^ and *RpL14*^*+/-*^. (A) Volcano plots showing differentially expressed transcriptional units (genes and transposons) in *RpL14*^*+/-*^ vs. *w*^*1118*^ (left) and *RpS17*^*+/-*^ vs. *w*^*1118*^ (right). (B) Heatmaps of genes significantly and differentially expressed between *RpS17*^+/-^ and *RpL14*^+/-^. Left: 230 transcriptional units more strongly upregulated in *RpS17*^*+/-*^. Right: 80 more strongly downregulated in *RpS17*^*+/-*^.(C) KEGG pathway enrichment (FDR < 0.05) of the 230 upregulated (left) and 80 downregulated (right) transcriptional units in *RpS17*^*+/-*^ relative to *RpL14*^*+/-*^.

To understand the molecular basis of the threshold effect observed in RP gene haploinsufficiency, we performed RNA-seq analysis of wing imaginal discs from wandering third instar larvae of *RpL14*^*+/-*^ (a sensory organ-non promoting *Minute*; No. 4 in Figure 1G) and *RpS17*^*+/-*^ (a sensory organ-promoting *Minute*; No. 7 in Figure 1G). Each was first compared to control *w*^*1118*^, and transcriptional changes were quantified. We identified 393 and 664 differentially expressed transcriptional units (genes and transposons) in *RpL14*^*+/-*^ and *RpS17*^*+/-*^, respectively, with a significance threshold of *p* < 0.01 (Figure 3A) (transcript lists in Supplemental Table 1A and 1B). In *RpL14*^*+/-*^, the number of upregulated (210) and downregulated (183) transcripts was nearly balanced (Figure 3A, left). In contrast, *RpS17*^*+/-*^ showed a skewed distribution, with more transcripts upregulated (454) than downregulated (210) (Figure 3A, right), suggesting a stronger transcriptional activation.

To identify candidates associated with the sensory organ-promoting phenotypes, we focused on transcripts that were differentially expressed between the two mutants. As shown in the heat maps (Figure 3B) (transcript lists in Supplemental Table 2A and 2B), 230 transcriptional units were significantly more upregulated in *RpS17*^*+/-*^ than *RpL14^+/-^* (left panel), while 80 transcriptional units were more strongly downregulated in *RpS17*^*+/-*^ (right panel) (see Methods for selection criteria).

KEGG pathway analysis of the 230 upregulated transcripts revealed four significantly enriched pathways in *RpS17*^*+/*-^ : (1) metabolism by cytochrome P450/Glutathione S-Transferases, (2) non-homologous end-joining, (3) biosynthesis of cofactors, and (4) pentose and glucuronate interconversions (Figure 3C). The 80 down-regulated transcripts were significantly enriched for pathways involved in (1) pentose phosphate pathway, (2) beta-alanine metabolism, (3) insect hormone biosynthesis, and (4) Toll and Imd signaling pathway (Figure 3C). These findings suggest that the RP gene haploinsufficiency above a critical severity, such as in *RpS17*^*+/-*^, triggers a stronger and coordinated stress response. This includes upregulation of detoxification, DNA repair, and metabolic remodelling pathways, while key anabolic, developmental, and innate immune pathways are sacrificed. Such a transcriptomic shift may underlie the sensory organ-promoting effect observed in *Minute* mutants.

### Upregulation of Xrp1 is required for extra sensory organ formation in minute mutants

**Figure 4.**
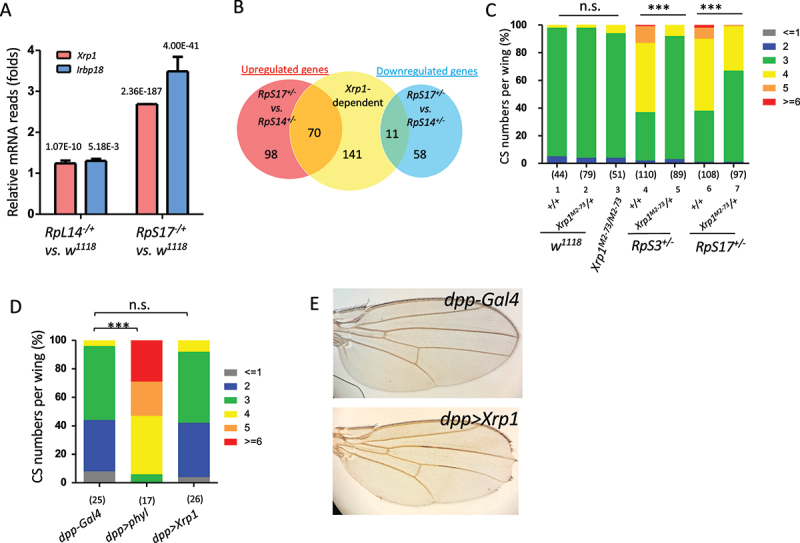
Xrp1 upregulation is required for ectopic cs formation in strong *minute* mutants. (A) Relative expression levels of *Xrp1* and *Irbp18* from RNA-seq data. *P*-values are indicated on the top of each column. (B) Numbers of differentially expressed genes between *RpS17*^*+/-*^ and *RpL14*^*+/-*^ that are Xrp1-dependent, based on previously identified targets [16]. (C) Introduction of one copy of null allele *Xrp1*^*M2-73*^ significantly suppresses ectopic cs formation in *RpS17*^*+/-*^ and *RpS3*^*+/-*^ mutants. *Xrp1* mutation itself does not influence cs numbers (comparing lane 1–3). (D) Overexpression of Xrp1 does not significantly alter cs organ numbers along vein iii. (E) Adult wing images showing wing margin loss induced by *Xrp1* overexpression under *dpp-Gal4*.

Given the stronger transcriptional stress responses observed in *RpS17*^*+/-*^, we hypothesized that the threshold-dependent promotion of extra sensory organs in *Minutes* is mediated by stress-responsive transcription factors. Supporting this idea, RNA-seq data revealed that *Xrp1* and its heterodimeric partner *Irbp18* are markedly upregulated in strong *Minutes*. Specifically, RNAseq data showed that *Xrp1* [16] expression was modestly increased in *RpL14*^*+/-*^ (24% increase; *p* = 1.07 × 10^− 1 0^), but dramatically elevated in *RpS17*^*+/-*^ (168% increase; *p* = 2.36 × 10^− 1 8 7^) (Figure 4A). Similarly, *Irbp18* mRNA was upregulated by 30% in *RpL14*^*+/-*^ (*p* = 5.18 × 10^− 3^) and by 249% in *RpS17*^*+/-*^ (*p* = 4 × 10^− 41^).

To determine whether these expression changes correspond to broader Xrp1 activity, we queried a published dataset of *Xrp1*-dependent genes in *RpS3*^*+/-*^ mutants [16]. Remarkably, 42% (*N* = 70 out of 168) of the differentially expressed, upregulated genes in *RpS17*^*+/-*^ relative to *RpL14*^*+/-*^ overlapped with this Xrp1-regulated set, as did 16% (*N* = 11 out of 69) of the downregulated genes (Figure 4B) (Xrp1-dependent genes highlighted in yellow in supplementary Table 2A and 2B). These data indicate that the Xrp1-Irbp18 heterodimer is differentially and more strongly activated in *RpS17*^*+/-*^ compared to *RpL14*^*+/-*^.

To test whether Xrp1 is required for the ectopic formation of sensory organs, we introduced a loss-of-function allele, *Xrp1*^*M2-73*^ [16], into *Minute* mutant backgrounds. Heterozygosity for *Xrp1*^*M2-73*^ significantly suppressed the extra CS phenotype in both *RpS17*^*+/-*^ and *RpS3*^*+/-*^ mutants (lanes 4–7 in Figure 4C). In contrast, CS numbers in *Xrp1*^*M2-73/M2-73*^ homozygotes and *Xrp1*^*M2-73/+*^ heterozygotes alone were comparable to wild-type control (lanes 1–3 in Figure 4C). These results demonstrate that elevated Xrp1 expression supports ectopic CS formation in strong *Minutes*.

To determine whether Xrp1 is sufficient to drive sensory organ formation, we overexpressed *Xrp1* using *dpp-Gal4*, which is active along wing vein III [40]. *dpp-Gal4* induced ectopic CS formation when sensory organ promoting gene *phyllopod* (*phyl*) was misexpressed (lane 2 in Figure 4D) [41]. In contrast, overexpressing *Xrp1* by *dpp-Gal4* did not significantly alter CS numbers in wings (Figure 4D), indicating that Xrp1 alone is not sufficient to induce ectopic CS formation. Consistent with it, any known regulators that are directly involved in neural fate determination or differentiation was not identified in the Xrp1 targets that are differentially upregulated in *RpS17*^*+/-*^ vs. *RpL14*^*+/-*^ (Supplemental Table 2A). These results suggest that neuronal fate is influenced indirectly by Xrp1. With its known role in inhibiting growth and survival of imaginal disc cells [16,42], Xrp1 overexpression caused wing margin loss (Figure 4E). Thus, while elevated Xrp1 is required, additional *Minute*-specific contexts may be required for promoting sensory organ fate.

## Discussion

In this study, we reveal a novel compensatory neurogenic mechanism under severe ribosomal stress, thus expanding the functional repertoire of the ribosome stress response. The classical Ac/Sc-dependent pathway for sensory organ formation serves as a powerful model to study neurogenesis and neural fate determination. Our genetic analyses reveal that RP gene haploinsufficiency has dual and seemingly opposite effects on sensory organ formation, depending on its severity. The strong Minutes in group III, which cause stronger bristle shortening and prolonged larval development, promote the formation of extra sensory organs. In contrast, RP mutations with non-, weak-, or intermediate-bristle shortening phenotypes further reduced microchaete numbers when combined with *ac sc* hypomorphic allele. Since non-*Minute* also reduces microchaete numbers, these data strongly support the hypothesis that the reduction is generally caused by RP gene heterozygous, not by RP gene haploinsufficiency. Together, these sensitive genetic interactions suggest that the maximal ribosome levels are crucial for proneural protein-driven neural precursor determination or differentiation.

Previous analyses of *RpS3* allelic series demonstrated that bristle shortening is proportional to the reduction of *RpS3* mRNA [11]. Our results, here, show that the formation of extra sensory organs occurs only in strong *Minute* mutants, those with bristle shafts shortened by at least 30%. This observation supports a model in which RP gene haploinsufficiency influences sensory organ development through two distinct mechanisms: (1) bristle length is gradually reduced in proportion to the loss of ribosome function or abundance. (2) ectopic sensory organ formation is triggered when ribosomal stress crosses a critical threshold, likely via a Xrp1-dependent mechanism.

Xrp1 is a key regulator of the cellular stress response and is essential for inducing changes associated with cell competition in *Minute* mutants. However, our transcriptomic analysis reveals striking differences in the degree of *Xrp1* activation across *Minute* genotypes. In *RpL14*^*+/-*^ mutants, which display a 23% reduction in bristle shaft length, *Xrp1* levels increased by only 24%. In contrast, *RpS17*^*+/-*^ mutants, which exhibited a 30% reduction in shaft length, showed a dramatic 168% upregulation of *Xrp1*. This marked difference in Xrp1 induction (24% vs. 168%) suggest *Minute* mutations vary in their capacity to activate Xrp1. Given that high Xrp1 levels impair translation and reduce cell viability [16,42], we propose that strong Xrp1 upregulation is induced only under severe ribosome stress.

Although Xrp1 is required for the formation of extra sensory organs in strong *Minutes*, its function is clearly context-dependent. Neither *Xrp1* loss-of-function mutants nor wings overexpressing *Xrp1* alone exhibited changes in CS number, indicating that Xrp1 is neither sufficient nor strictly necessary for Ac/Sc-dependent neurogenesis. Supporting this, only 42% and 16% of the genes differentially upregulated and downregulated, respectively, between strong and moderate *Minute* mutants are regulated by Xrp1. This suggests that additional, Xrp1-independent mechanisms contribute to the sensory organ patterning changes observed under ribosomal stress.

## Supplementary Material

Supplemental Material

## Data Availability

All data, including the RNAseq results of the selected genes are available within the figures and the supplementary tables.
